# Trace element-linked DNA methylation sites and their association with type 2 diabetes and cardiovascular diseases: EPIC-Potsdam cohort study

**DOI:** 10.1186/s13148-025-01991-0

**Published:** 2025-10-16

**Authors:** Barbaros Eroglu, Fabian Eichelmann, Olga Kuxhaus, Anna P. Kipp, Tanja Schwerdtle, Hajo Haase, Lutz Schomburg, Matthias B. Schulze

**Affiliations:** 1 Department of Molecular Epidemiology, German Institute of Human Nutrition Potsdam-Rehbruecke, 14558 Nuthetal, Germany; 2 TraceAge – DFG Research Unit on Interactions of Essential Trace Elements in Healthy and Diseased Elderly, Potsdam-Berlin-Jena-Wuppertal, Germany; 3 German Center for Diabetes Research (DZD), Munich-Neuherberg, Germany; 4 Institute of Nutritional Science, University of Potsdam, Nuthetal, Germany; 5 Department of Nutritional Physiology, Institute for Nutritional Sciences, Friedrich-Schiller University Jena, Jena, Germany; 6 Max Rubner-Institut, Federal Research Institute of Nutrition and Food, Karlsruhe, Germany; 7 Department of Food Chemistry and Toxicology, Technische Universität Berlin, Straße Des 17. Juni 135, 10623 Berlin, Germany; 8 Institute for Experimental Endocrinology, Charité-Universitätsmedizin Berlin, Berlin, Germany

**Keywords:** Trace elements, DNA methylation, EWAS, Type 2 diabetes, Cardiovascular disease

## Abstract

**Background:**

The trace elements (TEs) selenium, zinc, copper, manganese, iodine and iron are essential micronutrients that support essential metabolic functions. Imbalance in their homeostasis might contribute to the pathogenesis of major age-related chronic diseases including type 2 diabetes (T2D) and cardiovascular diseases (CVD). Emerging evidence suggests that TEs may affect health outcomes via epigenetic changes. However, few epigenome-wide association studies (EWAS) have explored TE-associated DNA methylation markers and their links to chronic disease outcomes.

**Methods:**

We conducted TE-specific exploratory EWAS using a random subcohort (n = 1030) from the European Prospective Investigation into Cancer and Nutrition (EPIC)-Potsdam cohort. The association between identified CpG sites and incident chronic diseases was evaluated using a case-cohort design comprising random subcohort participants and incident cases of T2D (n = 654) and CVD (n = 334). DNA methylation was measured with the MethylationEPIC BeadChip array. We used Prentice-weighted Cox proportional hazards regression models to estimate multivariable-adjusted hazard ratios (HRs) and 95% confidence intervals (CIs) for the associations of TE-associated CpG sites with each incident chronic disease.

**Results:**

In the copper EWAS, we identified six CpG sites (cg00398673, cg03957124, cg05736499, cg07573872, cg11503550, and cg18513344) that were significantly associated with serum copper concentrations with a False Discovery Rate < 0.05. All associated CpG sites showed lower methylation levels in association with higher serum copper levels. Higher methylation levels of cg00398673 (HR per SD: 0.74, 95% CI 0.63–0.88), cg03957124 (HR per SD: 0.52, 95% CI 0.41–0.66), cg05736499 (HR Q5 vs Q1: 0.25, 95% CI 0.14–0.47), and cg18513344 (HR Q5 vs Q1: 0.37, 95% CI 0.24–0.57) were associated with decreased risk of developing T2D, and higher methylation levels of cg07573872 were associated with decreased risk of developing CVD (HR per SD: 0.85, 95% CI 0.72–0.99). We did not observe any CpG sites that were significantly associated with other TEs.

**Conclusions:**

Serum copper levels are inversely associated with a number of CpG sites. Methylation levels at these CpG sites were inversely associated with developing T2D and CVD. These findings may provide new insights on understanding the increased risk of T2D and CVD with elevated blood copper levels.

**Supplementary Information:**

The online version contains supplementary material available at 10.1186/s13148-025-01991-0.

## Introduction

The trace elements (TEs) copper (Cu), iron (Fe), iodine (I), manganese (Mn), selenium (Se) and zinc (Zn) are essential micronutrients that are required in minute quantities for normal functioning of the human body. They play crucial roles in various processes such as enzymatic reactions, immune response, and nutrient metabolism among other physiological functions. An imbalance of TE levels can contribute to the pathogenesis of major age-related chronic diseases including type 2 diabetes (T2D), cardiovascular diseases (CVD) and cancer [1]. For example, blood TE levels were associated with incident T2D, CVD and colorectal cancer in the European Prospective Investigation into Cancer and Nutrition (EPIC)-Potsdam cohort, as reported recently [2].

Emerging evidence suggests that TEs may be linked to disease risk via their effects on epigenetic regulation [3]. DNA methylation is an epigenetic mechanism that involves the addition of methyl groups to the 5′ carbon of cytosine-phosphate-guanine (CpG) dinucleotide sequences in the genome, without changing the underlying DNA sequence [4]. It plays a pivotal role in gene expression, and abnormalities in DNA methylation are associated with a number of diseases including T2D [5–7], CVD [8] and cancer [9]. DNA methylation is susceptible to environmental factors [10, 11], including supply of TEs [12]. Zn is an important co-factor of epigenetically active enzymes such as histone deacetylases and DNA methyltransferases, and Zn deficiency has been associated with global DNA hypermethylation [13]. Cu interferes with expression and activity of the enzyme *S*-adenosylhomocysteine hydrolase which can affect DNA methyltransferase activity and expression (14).

Besides their effects on global epigenetic processes, TEs have also been linked to site-specific epigenetic modifications. An epigenome-wide association study (EWAS) investigated the associations between plasma Cu levels and DNA methylation and identified four novel Cu-associated CpG sites, one of which was associated with an increased risk of acute coronary syndrome [15]. Another EWAS conducted in Dongfeng-Tongji cohort identified six CpG sites associated with plasma Zn levels, two of which were associated with incident risk of lung cancer [16]. Although few other EWASes have investigated the association between TEs and DNA methylation, they were not conducted in blood but in placenta [17, 18] or in cord blood [19, 20], limiting their generalizability to adult populations. Together, these findings emphasize the importance of investigating how TE-associated DNA methylation changes contribute to disease risk. To the best of our knowledge, no EWAS has investigated the effect of multiple TEs on DNA methylation and further investigated the effects of identified CpG sites on T2D and CVD risk. In addition, most prior studies utilized the Illumina Infinium HumanMethylation450 BeadChip (450 K), which measures DNA methylation at ~ 485,000 CpG sites, covering roughly half the number of sites compared to the more comprehensive Illumina Infinium MethylationEPIC BeadChip (850 k) array that assesses DNA methylation at ~ 850,000 CpG sites.

In the present study, we conducted an EWAS within the EPIC-Potsdam cohort using a high-density array (850 k) to identify CpG sites that are associated with serum concentrations of TEs and two functional biomarkers of TEs, namely selenoprotein P (SelenoP) and free zinc (Free Zn). Subsequently, we examined the associations between these identified CpG sites and the risk of developing T2D and CVD.

## Methods

### Study design and population

The EPIC-Potsdam study is a prospective cohort study that enrolled 27,548 participants (16,644 women and 10,904 men) from the general population of Potsdam, Germany, and its surrounding areas between 1994 and 1998 [21]. The baseline examination consisted of anthropometric measurements, blood sampling, a validated semi-quantitative food frequency questionnaire, and collection of information on prevalent diseases and sociodemographic and lifestyle characteristics. All study participants provided written informed consent prior to their involvement, and the study received approval from the ethics committee of the Medical Society of the State of Brandenburg [22]. Participants were contacted every 2–3 years in the course of active follow-up, with response rates over 90% for each follow-up round. Detailed information about recruitment and follow-up procedures is described elsewhere [22, 23].

**Fig. 1:**
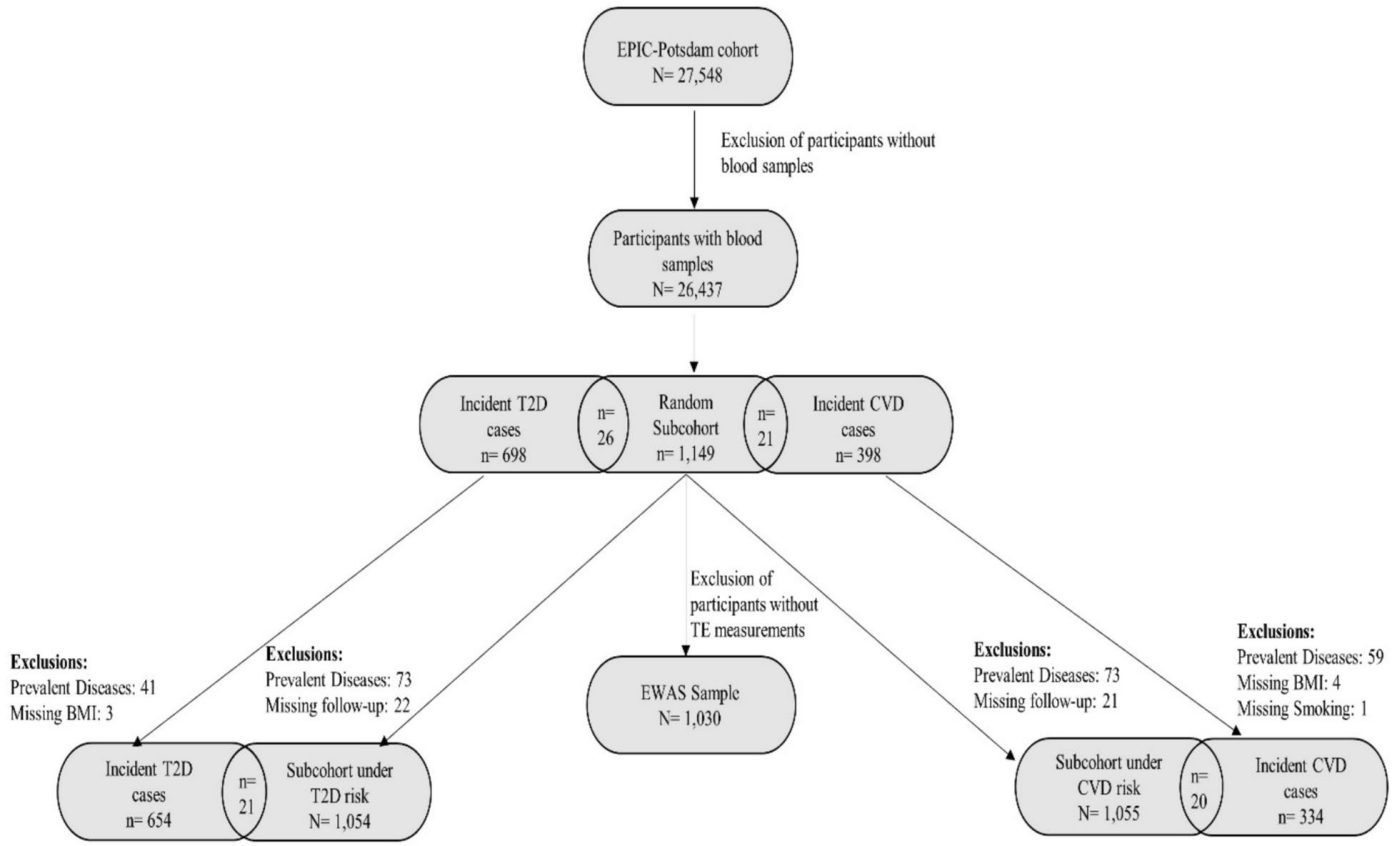
Flow chart of study sample derivation for EWAS and T2D and CVD analysis

A case-cohort study was conducted within the EPIC-Potsdam study to enable efficient molecular phenotyping while ensuring the generalizability of the results to the entire cohort [24]. DNA methylation was measured in a randomly selected subcohort of EPIC-Potsdam (n = 1149). The analytical sample for EWAS consisted of those subcohort participants with available serum TE measurements (n = 1030). The associations between identified CpG sites and incident chronic diseases were evaluated using the case-cohort design, comprising the aforementioned random subcohort of EPIC-Potsdam and all incident cases of T2D and CVD identified in the full cohort during follow-up until August 31, 2005, for T2D and November 30, 2006, for CVD. We excluded individuals from the case-cohort analyses if they had prevalent T2D or CVD at baseline, unclear disease status, missing covariate information, or lacked follow-up information. The analytical sample comprised 1687 participants for T2D including 654 cases (median follow-up time 6 years), and 1369 participants for CVD including 334 cases (median follow-up time 7.9 years) (Fig. 1).

### Outcome ascertainment

Incident diabetes cases were identified during follow-up through self-report of diagnosis, antidiabetic medication use or dietary treatment due to diabetes during follow-up. Additionally, death certificates and information from tumor centers, physicians, or clinics were screened for indications of incident diabetes. All incident cases were verified by questionnaires sent to the diagnosing physician asking about the date and type of diagnosis, diagnostic tests, and treatment of diabetes. Only physician-verified cases with a diagnosis of T2D (ICD-10 code E11), and the cases diagnosed after the baseline examination, were included as incident cases.

Incident CVD was defined as incidence of primary nonfatal and fatal myocardial infarction and stroke (International Statistical Classification of Diseases and Related Health Problems, Tenth Revision [ICD-10]) code: I21 for acute myocardial infarction (MI), I63.0 to I63.9 for ischemic stroke, I61.0 to I61.9 for intracerebral and I60.0 to I60.9 for subarachnoid hemorrhage, and I64.0 to I64.9 for unspecified stroke). Incident CVD cases were identified through self-report in follow-up questionnaires or information from the death certificates. Only physician-verified cases were included in the analysis. Total CVD cases were calculated by combining the verified incident cases of MI and stroke depending on whichever occurred first.

### Baseline characteristics

Sociodemographic and lifestyle characteristics were collected using self-administered and interviewer-based questionnaires at recruitment [21]. These included information on smoking, leisure time physical activity, medication use, educational attainment and alcohol consumption. Anthropometric measurements of participants were obtained by trained personnel at baseline. BMI was calculated as weight (kg) divided by the square of the height (m). Hypertension was defined as systolic BP ≥ 140 mmHg, diastolic BP ≥ 90 mmHg, self-reported hypertension diagnosis, or use of anti-hypertensive medication. Information on the amount and frequency of food and beverage intake, and supplement use was collected at baseline via validated food frequency questionnaire described previously [25]. A diet score (based on the Mediterranean diet score adapted to non-Mediterranean populations) was computed to take into consideration overall healthy diet as a determinant of TE status. Construction of this score has been documented elsewhere [26].

### Measurement of TE profiles

A sample of 30 mL peripheral blood from each participant was obtained by trained personnel at baseline. Blood was separated into serum, plasma and blood cells and stored in tanks of liquid nitrogen at − 196 °C or in deep freezers at − 80 °C until time of analysis. TEs (Cu, Fe, I, Mn, Se, Zn) and their functional biomarkers (SelenoP and Free Zn) were measured in serum. For the measurement of TEs we used the method published previously [27]. Briefly, 50 µL of sample was diluted with 440 µl of a diluent solution. As internal standard and for isotope dilution analysis 10 µL of a solution containing 50 µg/L ^77^Se and 5 µg/L Rh was added to give a total volume of 500 µL. This solution was directly subjected to analysis via inductively coupled plasma tandem mass spectrometry (ICP-MS/MS) (Agilent ICP-QQQ-MS 8800, Agilent Technologies, Waldbronn, Germany). SelenoP concentrations were measured using validated sandwich ELISA (selenOtest ELISA, selenOmed GmbH, Berlin, Germany), described in detail previously [28], and free Zn concentrations were measured by a Zinpyr-1-based fluorometric microassay as described before [29].

### DNA methylation analysis

DNA extracted from the peripheral blood of the participants taken at baseline was used to measure DNA methylation with the Illumina 850 k array. Raw.idat files were processed and functionally normalized using the meffil R package [30]. We excluded any sample in which more than 10% of CpG sites exhibited low bead counts or failed detection (*p* > 0.1), and we removed CpGs for which these metrics were poor in over 10% of samples. Additional sample‐level QC discarded individuals with sex‐prediction outliers (> 5 SD from expected), overall genotype concordance below 0.80, and probes with SNP‐genotype concordance below 0.95 or with methylated versus unmethylated intensities exceeding ± 5 SD. Cell‐type proportions were estimated from whole‐blood data using the method described by Reinius et al. [31]. Samples were randomly allocated across plates, and no technical replicates were included. Beta values were transformed to M-values using Logit transformation to increase statistical validity of the analyses [32]. Surrogate variables to control for batch effects at the modeling stage were generated with SmartSVA [33].

### Statistical analysis

Demographics and laboratory characteristics were summarized as median with interquartile range (IQR) for continuous variables and percentages for categorical variables. For Mn, 122 concentration values were below the limit of detection (LOD), and 151 concentration values were below the limit of quantification (LOQ) among participants included in EWAS sample. Left-censored data were handled by substituting by LOD/√2 for censored values less than LOD and by LOQ/√2 for censored values less than LOQ.

To explore the associations between serum TE levels and methylation levels at CpGs, we conducted linear regression, adjusting for age, sex, BMI, alcohol consumption, smoking, diet quality (assessed by the Mediterranean Diet Score), education, the proportions of major leukocytes (CD4 + T cells, CD8 + T cells, NK cells, B cells, monocytes, and granulocytes) and surrogate variables generated from SmartSVA. Concentrations of TEs were log-transformed to approximate normal distribution prior to analyses. Benjamini–Hochberg false discovery rate (FDR) [34] was applied to correct for multiple testing; FDR < 0.05 was applied as the genome-wide significance cut-off.

To investigate the associations between baseline TE-associated CpG sites and risk of incident T2D and CVD, Prentice-weighted Cox proportional hazards models were used, to account for the case-cohort design, stratified by age at recruitment [24]. M-values of CpG sites identified in the TE-EWAS were Z-standardized (mean = 0, SD = 1) to allow comparison of strength of the association across CpG sites. Linearity of the relationships was examined by restricted cubic splines, with three knots fitted at the 10th, 50th and 90th percentile of M-value distribution, and the cubic spline and linear models were compared using likelihood ratio test. The associations that deviated from linearity were assessed categorically, according to the quintiles of M-values of CpG sites in the subcohort. We evaluated the Schoenfeld residuals to validate the appropriateness of the proportional hazards assumption. Dependent time variable was defined as the time period between the age of recruitment and the age of exit (age of diagnoses or age of death or censoring). The first model was adjusted for age (stratified in years), sex, BMI (continuous; kg/m^2^), alcohol consumption (non-drinker [lifetime non-user and former user], low [≤ 6 g/day], moderately low [>6 to ≤ 12/g/day], moderately high [>12 to ≤ 24/g/day], high [>24 to ≤ 60 g/day], very high [> 60 g/day]), smoking (never smoker, former smoker, current smoker < 20 cigarettes/day, current smoker ≥ 20 cigarettes/day) and blood cell type proportions. The second model was further adjusted for education (no degree/vocational training, trade/technical school, university degree), leisure time physical activity (continuous; defined as the sum of sports, biking and gardening in hours per week), prevalent hypertension (yes or no), anti-hypertensive medication use (yes or no), lipid-lowering medication use (yes or no), vitamin and mineral supplement use (yes or no) and dietary quality (continuous; assessed by the Mediterranean score).

All analyses were performed using R (version 4.3.0) statistical software. All reported P values are two-sided.

### Pathway analysis

To investigate the biological pathways linking TEs and DNA methylation, we performed pathway enrichment analysis using the Kyoto Encyclopedia of Genes and Genomes (KEGG) genelists and the gometh function from the missMethyl package (version 1.38.0) [35]. Pathways with an FDR corrected *p* value < 0.05 were considered to have a significant association.

### Mediation analysis

The associations between TEs and T2D and CVD risk were previously demonstrated in the EPIC-Potsdam cohort [2]. We further investigated the potential mediating role of TE-associated CpG sites with T2D and CVD risk. For the mediation analyses, we only investigated the mediation effect if both the exposure and the proposed mediator were associated with the outcome, fulfilling the predefined mediation criteria [36]. We estimated the proportion explainable (PE) as percentage attenuation of the association between TE and disease risk using Cox models with adjustment for TE-associated CpG sites, and comparing the same model without TE-associated CpG site adjustment. PE and the corresponding 95% CI were estimated using a bootstrapping procedure with 1000 replications.

## Results

### Baseline characteristics

Baseline characteristics of the EPIC-Potsdam subcohort participants are presented in Table 1. The median age of participants was 50.9 years (IQR: 42.5–57.9), with a higher proportion of women (60%). Median BMI was 25.6 kg/m^2^ (IQR: 23.2–28.2), and approximately half of the participants were never smokers. Median leisure time physical activity was 5.0 h/w (IQR: 2.5–8.0), and the majority of participants had secondary education or higher.

**Table 1:** Baseline characteristics of EPIC-Potsdam subcohort

Characteristic	N = 1030
Age	50.9 (42.5, 57.9)
Female	621 (60.3%)
BMI (kg/m2)	25.6 (23.2, 28.2)
Physical Activity (h/week)	5.0 (2.5, 8.0)
Education Level (%)	
Primary School	405 (39.3%)
Secondary School	241 (23.4%)
College/Higher	384 (37.3%)
Smoking Status	
Never	492 (47.8%)
Former Smoker	312 (30.3%)
Current Smoker (< 20 d)	164 (15.9%)
Current Smoker (≥ 20 d)	62 (6.0%)
Alcohol Intake	
None	26 (2.5%)
Low	409 (39.8%)
Moderately Low	204 (19.8%)
Moderately High	202 (19.6%)
High	165 (16.0%)
Very High	24 (2.3%)
Mediterranean Diet Score	9.0 (7.0, 11.0)
Prevalent Hypertension	526 (50.5%)
Anti-hypertensive Medication Use	215 (20.8%)
Lipid Lowering Medication Use	60 (5.8%)
Use of Vitamin Supplement	168 (16.3%)
Use of Mineral Supplement	108 (10.4%)
Copper [µg/L]	1018 (876, 1192)
Iron [µg/L]	929 (721, 1154)
Iodine [µg/L]	56.2 (49.3, 65.0)
Manganese [µg/L]	1.0 (0.5, 1.6)
Selenium [µg/L]	79.9 (71.5, 90.4)
Zinc [µg/L]	732 (648, 831)
Free Zinc [nM]	0.6 (0.4, 0.7)
Selenoprotein P [mg/L]	5.3 (4.5, 6.3)

Data are expressed as medians (IQR) for continuous variables and n (%) for categorical variables

### TE-associated DNA methylation

**Fig. 2:**
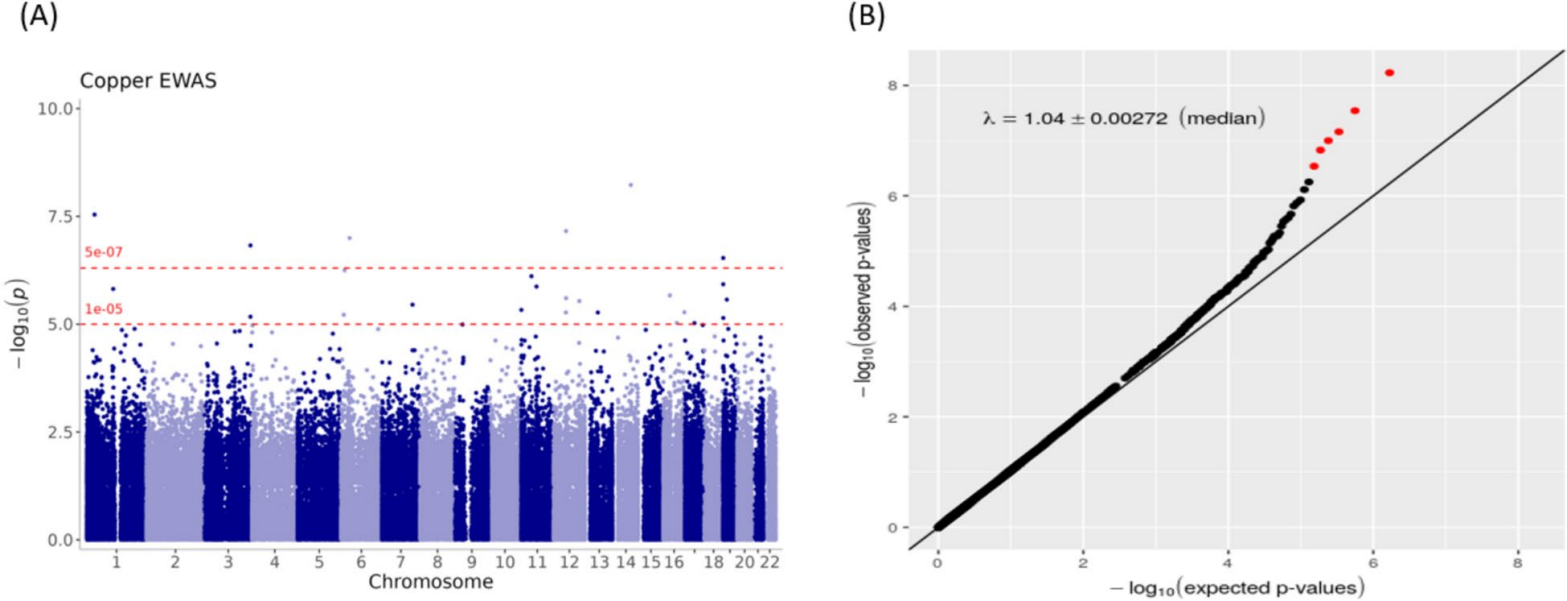
Manhattan Plot (**A**) and Q-Q Plot (**B**) for EWAS results for serum Cu levels. The x-axis indicates genomic locations of the CpG sites; the y-axis indicates − log10 (*p* value) of the associations. The red dashed line at the top corresponds to the false discovery rate (FDR) = 0.05 threshold; the red dashed line at the bottom represents suggestive association (*p* value < 1e−05). In the Q–Q plot, the *x*-axis shows the expected − log10 (*p* value), whereas the *y*-axis indicates the observed − log10 (*p* value)

We identified six CpG sites in EWAS (cg00398673, cg03957124, cg05736499, cg07573872, cg11503550, and cg18513344) associated with serum Cu levels with an FDR < 0.05 after adjustment for age, sex, smoking, alcohol consumption, BMI, diet quality, education, blood cell type proportions and surrogate variables (Manhattan and Q-Q Plot, Fig. 2). Higher serum Cu levels were associated with lower methylation at all identified CpG sites (Table 2). Three of the six CpG sites (cg00398673, cg03957124, and cg11503550) were located within intergenic regions, and three were located within gene bodies of tensin 2 (*TNS2*; cg05736499), strawberry notch homolog 2 (*SBNO2*; cg07573872), and mucin 4 (*MUC4*; cg18513344).

**Table 2:** CpG sites associated with serum Cu levels among EPIC-Potsdam subcohort participants (n = 1030)

					Association with Cu		
CpG	Chromosome: Position	Gene	Region	Methylation Beta-value, Median (IQR)	*P* value	FDR	Estimate (SE)^a^
cg00398673	1:33,448,891			0.497 (0.473–0.523)	2.88E−08	0.012	−0.097 (0.017)
cg03957124^1^	6:37,016,869			0.481 (0.423–0.528)	1.00E−07	0.021	−0.109 (0.020)
cg05736499	12:53,445,115	*TNS2*	Body	0.446 (0.402–0.489)	6.93E−08	0.019	−0.114 (0.021)
cg07573872^1^	19:1,126,342	*SBNO2*	Body	0.773 (0.745–0.799)	2.91E−07	0.041	−0.173 (0.034)
cg11503550	14:75,867,017			0.533 (0.506–0.559)	5.92E−09	0.005	−0.122 (0.021)
cg18513344^1^	3:195,531,298	*MUC4*	Body	0.244 (0.216–0.276)	1.49E−07	0.025	−0.133 (0.025)

FDR: False Discovery Rate

^1^Included in Illumina Infinium HumanMethylation450 BeadChip

^a^Multiple linear regression models were used as log-transformed Cu as independent variable and M-values of CpG sites as dependent variables. Adjusted for age, sex, BMI, alcohol consumption, smoking, Mediterranean Diet Score, education, blood cell type proportions and surrogate variables. Estimate is the change in M-values per log-unit increase in Cu levels

We did not observe that other TEs, SelenoP or Free Zn were significantly associated with any CpG site after FDR correction. The list of CpG sites with suggestive epigenome-wide significance is provided in Supplementary Tables 1–8.

### Association of Cu-associated CpG sites with T2D risk

The associations between cg00398673, cg03957124 and cg11503550 with T2D risk were linear according to the spline regression analyses, while associations between cg18513344, cg05736499 and cg07573872 with T2D risk deviated from linearity (Supplementary Fig. 1).

In fully adjusted multivariable Cox regression models, higher methylation levels of cg00398673 (HR per SD: 0.74, 95% CI 0.63–0.88), cg03957124 (HR per SD: 0.52, 95% CI 0.41–0.66), cg05736499 (HR Q5 vs Q1: 0.25, 95% CI 0.14–0.47), and cg18513344 (HR Q5 vs Q1: 0.37, 95% CI 0.24–0.57) were associated with decreased risk of developing T2D (Table 3).

**Table 3:** Associations between CpG sites (Z-standardized M-values) and incident T2D, EPIC-Potsdam study

		Hazard ratio (95% CI) Type 2 Diabetes	
CpG site	No. of cases/Total	Model 1	Model 2
cg00398673	654/1687	0.73 (0.61–0.86)	0.74 (0.63–0.88)
cg03957124	654/1687	0.51 (0.40–0.65)	0.52 (0.41–0.66)
cg05736499			
Quintile 1	212/420	1 (reference)	1 (reference)
Quintile 2	141/346	0.65 (0.45–0.94)	0.69 (0.48–1.00)
Quintile 3	119/323	0.57 (0.38–0.85)	0.55 (0.36–0.84)
Quintile 4	99/307	0.50 (0.31–0.80)	0.46 (0.28–0.75)
Quintile 5	83/291	0.24 (0.13–0.45)	0.25 (0.14–0.47)
cg07573872			
Quintile 1	152/362	1 (reference)	1 (reference)
Quintile 2	124/326	0.74 (0.51–1.06)	0.74 (0.52–1.07)
Quintile 3	134/341	0.93 (0.64–1.36)	0.94 (0.56–1.38)
Quintile 4	118/329	0.65 (0.44–0.96)	0.69 (0.47–1.01)
Quintile 5	126/329	0.75 (0.50–1.12)	0.78 (0.52–1.16)
cg11503550	654/1687	0.88 (0.74–1.04)	0.90 (0.76–1.07)
cg18513344			
Quintile 1	242/440	1 (reference)	1 (reference)
Quintile 2	138/348	0.41 (0.29–0.58)	0.39 (0.27–0.56)
Quintile 3	115/323	0.44 (0.31–0.63)	0.45 (0.31–0.65)
Quintile 4	79/286	0.34 (0.23–0.50)	0.36 (0.25–0.53)
Quintile 5	80/290	0.37 (0.25–0.57)	0.37 (0.24–0.57)

M-values were Z-standardized (mean = 0, SD = 1)

Model 1: Adjusted for age, sex, BMI, smoking, alcohol consumption and blood cell type proportions, Model 2: Model 1 + education level, physical activity, prevalent hypertension, anti-hypertensive medication use, lipid-lowering medication use, vitamin and mineral preparation use and Mediterranean Diet Score

### Association of Cu-associated CpG sites with CVD risk

The associations between four CpG sites (cg00398673, cg03957124, cg07573872, and cg11503550) with CVD risk were linear according to the spline regression analysis, while associations between cg05736499 and cg18513344 deviated from linearity (Supplementary Fig. 2).

We observed lower risk of developing CVD (HR per SD: 0.85, 95% CI 0.72–0.99) for higher methylation at cg07573872 in fully adjusted multivariable Cox regression. No significant associations were observed for other CpG sites with the risk of developing CVD (Table 4). We further investigated the mediating effect of cg07573872 on the association between Cu and CVD risk. The mediation analysis did not suggest a mediating effect of cg07573872 on the association between Cu and CVD risk (PE: 1%, 95% CI: −3 to 5%).

**Table 4:** Associations between CpG sites (Z-standardized M-values) and incident CVD, EPIC-Potsdam study

		Hazard ratio (95% CI) Cardiovascular Disease	
CpG Site	No. of cases/Total	Model 1	Model 2
cg00398673	334/1369	0.95 (0.76–1.18)	0.98 (0.78–1.23)
cg03957124	334/1369	0.76 (0.58–1.00)	0.79 (0.59–1.05)
cg05736499			
Quintile 1	65/274	1 (reference)	1 (reference)
Quintile 2	62/271	1.09 (0.69–1.72)	1.14 (0.71–1.82)
Quintile 3	79/279	1.37 (0.84–2.24)	1.47 (0.89–2.44)
Quintile 4	77/284	1.27 (0.73–2.20)	1.39 (0.79–2.45)
Quintile 5	51/261	0.78 (0.40–1.52)	0.89 (0.45–1.77)
cg07573872	334/1369	0.84 (0.72–0.98)	0.85 (0.72–0.99)
cg11503550	334/1369	1.01 (0.83–1.24)	1.03 (0.84–1.26)
cg18513344			
Quintile 1	78/277	1 (reference)	1 (reference)
Quintile 2	71/283	0.94 (0.62–1.42)	0.94 (0.61–1.45)
Quintile 3	61/269	0.92 (0.60–1.41)	0.96 (0.62–1.50)
Quintile 4	66/272	1.09 (0.71–1.68)	1.16 (0.75–1.78)
Quintile 5	58/268	1.01 (0.63–1.62)	1.07 (0.66–1.74)

M-values were Z-standardized (mean = 0, SD = 1)

Model 1: Adjusted for age, sex, BMI, smoking, alcohol consumption and blood cell type proportions, Model 2: Model 1 + education level, physical activity, prevalent hypertension, anti-hypertensive medication use, lipid-lowering medication use, vitamin and mineral preparation use and Mediterranean Diet Score

### Pathway analysis

In the pathway analysis of the 25 CpG sites showing suggestive associations with Cu levels (*P* < 1.0 × 10 − 5), we did not observe any significant pathways after FDR correction (Supplementary Table 9).

## Discussion

In this population-based EPIC-Potsdam cohort study, EWAS revealed that higher serum Cu levels were associated with decreased methylation at six CpG sites that had not been previously reported. Higher methylation at four of these sites was associated with reduced risk of T2D, and higher methylation at one site was associated with lower CVD risk in fully adjusted models. We did not observe any significant associations between other TEs, SelenoP and Free Zn with DNA methylation.

A previous EWAS of 1243 Chinese individuals identified four CpG sites associated with circulating Cu levels [15], however, without overlap with the six CpG sites that were identified in this study to be significantly associated with serum Cu levels. In the Dongfeng-Tongji cohort, an EWAS involving 1,689 individuals identified six CpG sites associated with plasma Zn levels [16]. In contrast, we did not observe any significant associations between serum Zn levels and DNA methylation. Several factors could explain the discrepancies of our results compared to those of previous EWAS. Firstly, participants in the EPIC-Potsdam study predominantly have German ancestry, while previous studies involved Chinese populations. Differences in DNA methylation profiles across ethnic groups are well-documented [37, 38]. Secondly, methodological differences could play a role; the previous EWAS on Cu [15] used the 450 K array, which measures methylation at approximately 485,000 CpG sites. In contrast, we employed the newer 850 k array, which covers nearly twice as many CpG sites, potentially allowing for the detection of associations not identified by earlier methods. Three of the six Cu-associated CpG sites (cg03957124, cg07573872, and cg18513344) identified in our study were not covered in the 450 k array. To our knowledge, this study is the first EWAS that investigated the associations between DNA methylation and TEs besides Cu and Zn, and TE biomarkers SelenoP and Free Zn using blood samples from a general population.

Cu is an essential metal for humans; however, excess levels can contribute to formation of reactive oxygen species, leading to oxidative damage and cell death [39]. A recent meta-analysis of longitudinal studies observed that higher levels of circulating Cu are associated with higher risk of CVD incidence [40]. In the EPIC-Potsdam study—also part of the meta-analysis—higher serum Cu levels were associated with an increased risk of CVD. A similar trend was observed for incident T2D; however, the association was not statistically significant.

Our findings support the hypothesis that a molecular mechanism contributing to the observed association of Cu with health outcomes may be driven by its ability to alter DNA methylation. Cu has been shown to inhibit the activity of *S*-adenosylhomocysteine hydrolase [14], a key enzyme that regulates the amount of S-adenosyl-methionine available for methylation reactions. Previous studies demonstrated that both higher intake and higher serum levels of Cu were associated with reduced levels of global DNA methylation [17, 41]. Besides effects of Cu on global methylation, our results indicate that site-specific methylation might play a role as well. Higher methylation at four Cu-associated CpG sites was associated with decreased T2D risk, independent of age, sex and other lifestyle risk factors. cg00398673 is located in an intergenic region and has not been reported in any EWAS result before to our knowledge. Therefore, its mechanistic role in T2D development is unknown. We replicate findings for cg03957124 (intergenic region), cg05736499 (*TNS2*), and cg18513344 (*MUC4*) that were previously reported to be associated with incident T2D in the basic model in an EWAS of around 18,000 Scottish individuals [42]. After adjusting for other risk factors, cg03957124 and cg18513344 were still significantly associated with incident T2D risk. The discrepancies between our findings and previous reports may be attributable to differences in methodological approaches, including the inclusion of medication use and diet quality in covariate sets.

The strongest associations we observed between Cu-associated CpG sites and T2D risk were for cg05736499 (*TNS2*) and for cg18513344 (*MUC4*). *TNS2* encodes C1-Ten, a protein tyrosine phosphatase that dephosphorylates insulin receptor substrate 1 (IRS-1), a critical mediator in insulin signaling that regulates glucose uptake and metabolism [43]. Decreased IRS-1 activity contribute to reduced glucose uptake and impaired insulin action [44]. In mice, inhibition of C1-Ten activity improved glucose tolerance by activating IRS-1 and AMP-activated protein kinase pathways [45]. Furthermore, high glucose conditions elevate C1-Ten expression, and this upregulation accelerates podocyte hypertrophy, a prominent feature of diabetic kidney disease [46]. *MUC4* encodes a membrane‐associated mucin involved in epithelial protection and maintaining the integrity of the gut barrier. Prior studies have largely focused on its role in inflammation [47], cancer [48] and epithelial homeostasis [49], rather than diabetes. Chronic low‐grade inflammation and altered epithelial function are common features of obesity and diabetes [50]. Furthermore, alterations in intestinal permeability may lead to an increased presence of infectious agents and dietary antigens in bloodstream, which can trigger immune reactions that subsequently cause damage to pancreatic beta cells [51]. In addition, differentially methylated CpGs at *MUC4* were observed between insulin resistant and insulin sensitive individuals [52]. Taken together, these findings raise the possibility that the changes in DNA methylation may be one of the mechanisms by which Cu influences T2D risk.

Previous EWASes have shown that higher methylation at five of the six identified CpG sites related to Cu (cg03957124, cg05736499, cg07573872, cg11503550, cg18513344) was associated with decreased CRP levels [42, 53], a well-established marker of inflammation. However, it remains unclear whether DNA methylation causally drives CRP changes, whether CRP influences DNA methylation, or whether both reflect a shared inflammatory process. Two Mendelian randomization studies investigated whether this association is causal or consequential, yet their findings remained inconclusive [53, 54]. Since the causal direction between DNA methylation and CRP levels remains unclear, we cannot exclude the possibility that chronic inflammation could play a role in the pathway between DNA methylation with T2D and CVD, in which case adjusting for CRP could introduce bias into our analyses. Thus, we did not include CRP levels in our adjustment sets.

Higher methylation at cg07573872 in the *SBNO2* gene was associated with decreased risk of CVD. This confirms previous observations that a higher methylation at this CpG site was associated with decreased risk of stroke and heart disease [42]. *SBNO2* is a negative regulator of IL-6 signaling, and it is upregulated in astrocytes as a direct response to cytokines and associated with increased neuroinflammation and neurodegeneration [55]. Considering the role of inflammation in stroke pathogenesis [56], this association could be one potential biological mechanism through which elevated Cu levels are linked to increased CVD risk.

Our study has several strengths. First, our study investigated a broad range of TEs (six), SelenoP and Free Zn in relation to DNA methylation. Second, the prospective design of the study allowed us to investigate whether the identified CpG sites are associated with T2D or CVD risk. Third, we employed the 850 k array, thus covering almost twice as many CpGs compared to the 450 K array which has been used by most previous EWAS [15].

Several limitations of this study should be mentioned. First, we measured serum TE levels and DNA methylation levels at the same time point; therefore, we cannot draw any conclusions about causality or the direction of the effect. Second, we measured DNA methylation in whole blood, which might not reflect important changes in disease-relevant tissues; however, Elliott et al. showed substantial correlation of T2D-associated CpG site methylation in whole blood with disease-relevant tissues such as adipose tissue, liver and pancreas [57]. Third, CVD in EPIC-Potsdam is defined as a composite of primary MI and stroke. Further research is needed, to address associations with less acute outcomes such as angina pectoris and arterial fibrillation. Fourth, we adjusted our analyses for potential confounders to eliminate confounding; however, we cannot rule out that residual confounding explains some of the observed associations. Lastly, our study sample consisted almost exclusively of participants with German ancestry; therefore, our findings may not be generalizable to other ethnicities, considering the epigenetic variations among different ethnic groups.

## Conclusions

In summary, we identified six novel CpG sites associated with serum Cu levels. Increased methylation at four of these CpG sites was associated with decreased risk of T2D incidence, while increased methylation at one CpG site was associated with decreased risk of CVD incidence. These results indicate that epigenetic alterations in relation to Cu status have a potential role in the development of cardiometabolic diseases.

## Supplementary Information

Additional file 1.

Additional file 2.

## Data Availability

The datasets analyzed in the current study are not publicly available due to data protection regulations. In accordance with German Federal and State data protection regulations, epidemiological data analyses of EPIC-Potsdam may be initiated upon an informal inquiry addressed to the secretariat of the Human Study Center (Office.HSZ@dife.de). Each request will then have to pass a formal process of application and review by the respective PI and a scientific board.

## Abbreviations

CpG: Cytosine-phosphate**-**guanine
CRP: C-reactive protein
CVD: Cardiovascular disease
EPIC: European Prospective Investigation into Cancer and Nutrition
EWAS: Epigenome-wide association study
FDR: False discovery rate
HR: Hazard ratio
ICP-MS/MS: Inductively coupled plasma tandem mass spectrometry
IL-6: Interleukin-6
IRS-1: Insulin receptor substrate 1
KEGG: Kyoto Encyclopedia of Genes and Genomes
LOD: Limit of detection
LOQ: Limit of quantification
PE: Proportion explainable
T2D: Type 2 diabetes
TE: Trace element
