# Supplementary material for: Trace element-linked DNA methylation sites and their association with type 2 diabetes and cardiovascular diseases: EPIC-Potsdam cohort study
Source: Clin Epigenetics. 2025 Oct 16;17:172. doi: 10.1186/s13148-025-01991-0 (PMC12529838; doi:10.1186/s13148-025-01991-0)
Supplement: Supplementary file 1 — Additional file 1. [file 13148_2025_1991_MOESM1_ESM.docx]

**Supplementary Figure 1.** Restricted cubic spline regression plots for T2D risk according to DNA methylation values of Cu-Associated CpG Sites


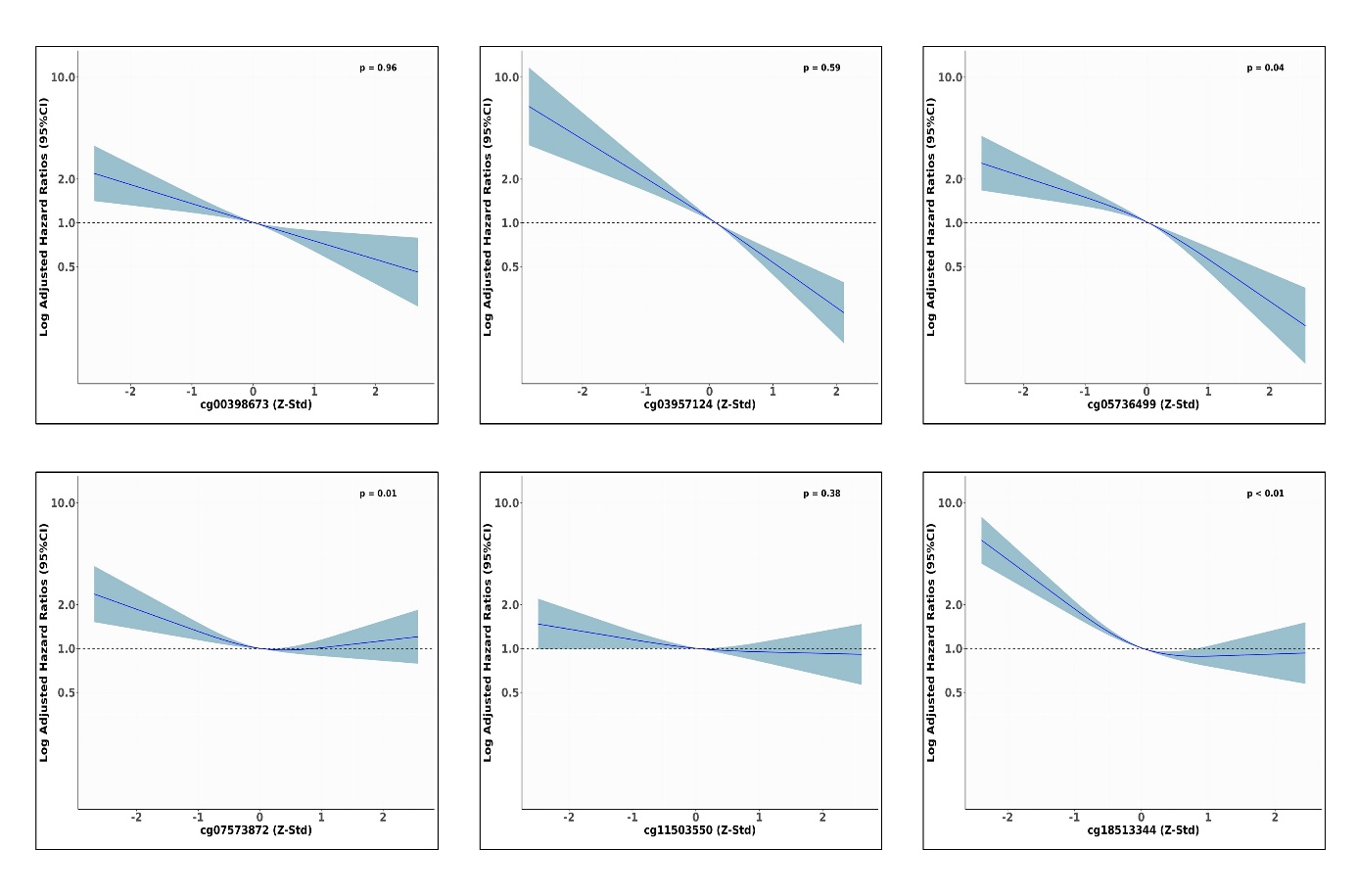


M-values were Z-standardized (mean = 0, SD = 1)

Models were adjusted for age, sex, BMI, smoking, alcohol consumption, education level, physical activity, prevalent hypertension, anti-hypertensive medication use, lipid-lowering medication use, vitamin and mineral preparation use and Mediterranean Diet Score and blood cell type proportions.

**Supplementary Figure 2.** Restricted cubic spline regression plots for CVD risk according to DNA methylation values of Cu-Associated CpG Sites

**
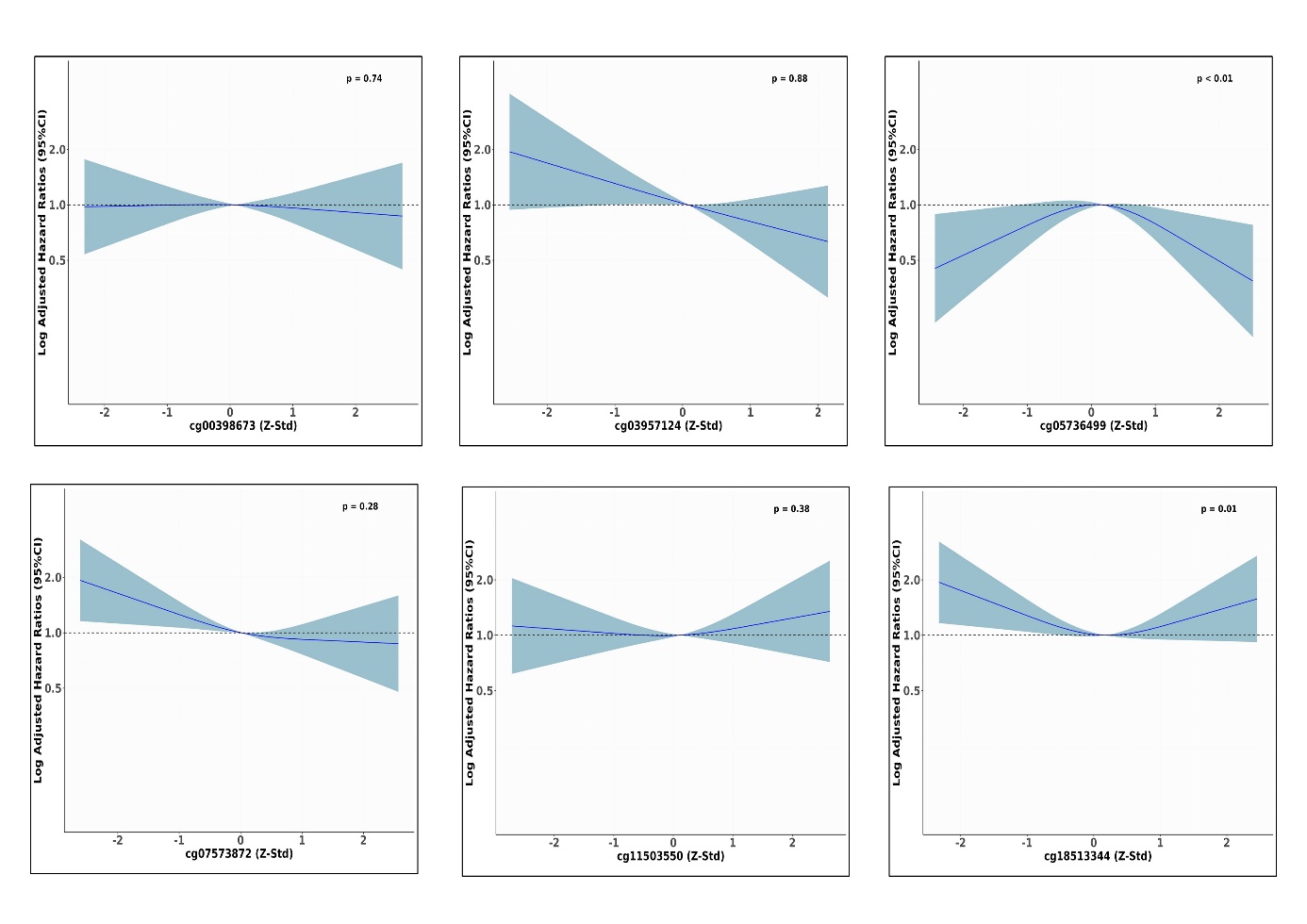
**

M-values were Z-standardized (mean = 0, SD = 1)

Models were adjusted for age, sex, BMI, smoking, alcohol consumption, education level, physical activity, prevalent hypertension, anti-hypertensive medication use, lipid-lowering medication use, vitamin and mineral preparation use and Mediterranean Diet Score and blood cell type proportions.
